# Street dancing enhances cognitive reserve in young females: an fNIRS study

**DOI:** 10.3389/fnins.2025.1640555

**Published:** 2025-10-02

**Authors:** Yongbo Wang, Quansheng Zheng, Yanbai Han, Yaqing Fan, Hongen Liu, Hongli Wang

**Affiliations:** ^1^College of Physical Education and Health, Guangxi Normal University, Guilin, China; ^2^College of General Education, Guizhou University of Commerce, Guiyang, China

**Keywords:** street dance, fNIRS, executive function, prefrontal cortex activation, cognitive flexibility

## Abstract

**Introduction:**

With the accelerating aging population, cognitive decline and dementia pose major public health challenges. Early intervention is crucial for mitigating these risks. Dance, with its high cognitive demands and multitasking coordination, has shown benefits for cognitive function. However, evidence on the effects of street dance on prefrontal cortex activation and executive function is limited. This study uses functional near-infrared spectroscopy (fNIRS) to explore how street dance impacts prefrontal activation and executive function, offering insights into early prevention of cognitive decline.

**Methods:**

A total of 28 healthy female college students were recruited and randomly assigned to a street dance intervention group (*n* = 14) or a control group (*n* = 14). The study was conducted between March and September 2024 at a university in Southwest China. The intervention group participated in an 18-week street dance program, three times per week, with each session lasting 80 min. The control group maintained their usual routines without structured physical activity. fNIRS was used pre- and post-intervention to assess changes in oxygenated hemoglobin (HbO₂) levels in the prefrontal cortex during the 2-back, Stroop, and More-Odd Shifting tasks.

**Results:**

Two-way repeated-measures ANOVA revealed significant Group × Time interaction effects in multiple prefrontal regions across the three tasks. In the 2-back task, the street dance group showed significant increases in HbO₂ in the right dorsolateral prefrontal cortex, accompanied by improvements in accuracy and faster reaction times. In the Stroop task, HbO₂ significantly increased in the right frontopolar and dorsolateral prefrontal cortices, reflecting enhanced inhibitory control. In the More-Odd Shifting task, significant activation was observed in the left inferior frontal gyrus, together with improved task-switching accuracy and reduced reaction times.

**Conclusion:**

The 18-week street dance intervention effectively improved working memory, inhibitory control, and cognitive flexibility, contributing to enhanced cognitive reserve. As a physical activity combining rhythm and coordination, street dance offers a promising early intervention strategy for delaying cognitive decline and reducing dementia risk.

**Clinical trial registration:**

https://www.chictr.org.cn/, identifier ChiCTR2400083689.

## 1 Introduction

With the accelerating trend of global aging, dementia has become a pressing public health challenge. Characterized by severe cognitive impairments, common types of dementia such as Alzheimer’s disease and vascular dementia not only significantly reduce patients’ quality of life but also place a heavy burden on families and society (World Health Organization [WHO], 2018). Studies have shown that pathological changes in dementia may begin accumulating 20–30 years before clinical symptoms appear, making early prevention and intervention a critical strategy for reducing its risk. Among these strategies, enhancing cognitive reserve is considered an essential means to delay cognitive decline (Stern, 2012; Livingston et al., 2020).

Cognitive reserve refers to an individual’s ability to enhance brain flexibility and compensatory capacity through education, occupational training, social activities, and cognitive stimulation, allowing for the maintenance of high levels of cognitive performance despite pathological changes (Savarimuthu and Ponniah, 2024; Stern and Barulli, 2019). The university stage represents a golden period for building cognitive reserve. Effective interventions during this period can not only improve individuals’ current learning and living conditions but also provide a scientific pathway for preventing future cognitive impairments and dementia (Stern et al., 2019; Winblad et al., 2016).

In recent years, physical activity has garnered significant attention as an effective method for enhancing cognitive reserve. Traditional aerobic and resistance training have been shown to improve attention, memory, and executive function by increasing cerebral blood flow, promoting neuroplasticity, and releasing neurotrophic factors (Contreras-Osorio et al., 2022; Xiong et al., 2021). Dance, as a unique form of exercise that combines movement memory, rhythm, and emotional expression, has demonstrated distinct advantages in improving cognitive function. Dance not only stimulates the prefrontal cortex to enhance cognitive flexibility but also increases the feasibility and engagement of interventions due to its enjoyable and interactive nature (Kattenstroth et al., 2013; Hackney et al., 2024).

Street dance originated in the United States in the 1970s, encompassing dance styles such as Breaking, Locking, and Popping that emerged from hip-hop culture. It is characterized by improvisation, strong rhythmicity, and expressive, coordinated movements. Among various types of dance, street dance has drawn attention for its complex movement transitions and diverse rhythmic variations. Street dance training requires participants to quickly adapt to changes in musical rhythm while completing high-intensity movement memory and coordination tasks, placing higher demands on attention, reaction speed, and executive function. This high level of cognitive load may significantly stimulate the prefrontal cortex, thereby promoting cognitive function (Shen et al., 2020; Zinelabidine et al., 2022). However, existing studies have predominantly focused on traditional physical activities and other forms of dance, with limited research on how street dance interventions improve cognitive function, particularly in terms of their impact on prefrontal neural mechanisms. Moreover, the potential of street dance interventions in enhancing cognitive reserve and preventing future cognitive impairments remains underexplored.

In this study, street dance is defined as an umbrella term for styles such as Breaking, Locking, and Popping, characterized by improvisation, rhythmic variety, and cognitively demanding choreographic sequences. This clear definition is adopted to distinguish street dance from other forms of dance or exercise and to clarify the rationale for its selection as an intervention.

Functional near-infrared spectroscopy (fNIRS) is a non-invasive brain imaging tool that provides crucial support for studying the neural mechanisms underlying physical activity interventions on cognitive function. By monitoring changes in oxygenated hemoglobin (△HbO_2_) concentration in real time, fNIRS can directly reflect the activation status of the prefrontal cortex, making it particularly suitable for exploring the neurobiological effects of exercise interventions (Herold et al., 2018; Llana et al., 2022) assessments to systematically analyze the effects of an 18-week street dance intervention on prefrontal activation and executive function in female college students. Additionally, it seeks to explore the potential mechanisms of street dance in enhancing cognitive reserve and reducing the risk of cognitive impairment.

The significance of this study lies not only in revealing the cognitive benefits of street dance interventions but also in elucidating their application potential in enhancing cognitive reserve and preventing cognitive decline. The findings of this research will provide a theoretical basis for designing scientifically informed physical activity intervention programs for college students. Furthermore, it will expand the application scenarios of dance interventions in the field of cognitive health, offering novel perspectives for the early prevention of cognitive impairments.

## 2 Materials and methods

### 2.1 Study design

This was a two-arm randomized controlled trial (RCT) with an intervention group and a control group, conducted between March and September 2024. The trial was registered at the Chinese Clinical Trial Registry on April 30, 2024, after the initiation of participant enrolment. Although registration occurred after recruitment began, the study protocol was established in advance and strictly followed, and all outcomes were fully reported in accordance with the CONSORT 2010 guidelines.

### 2.2 Participants

A total of 28 healthy adult female participants were recruited and randomly assigned to either the Street Dance Group (*n* = 14) or the Control Group (*n* = 14). At baseline, the Street Dance Group had a mean age of 20.07 ± 0.87 years and a mean BMI of 19.82 ± 1.91, while the Control Group had a mean age of 20.25 ± 1.41 years and a mean BMI of 20.41 ± 2.16. No significant between-group differences were observed in age or BMI (all *p* > 0.05). All participants were right-handed, had normal or corrected-to-normal vision, had no history of neurological or psychiatric disorders, and met the following inclusion criteria: (1) Age between 18.00 and 25.00 years; (2) Body Mass Index (BMI) within the normal range (18.50–24.90); (3) Healthy scalp with no head conditions, allowing for the use of near-infrared spectroscopy (fNIRS) equipment; (4) No prior experience with dance interventions within the past 3 months. Exclusion criteria: (1) History of neurological or psychiatric disorders (e.g., epilepsy, depression, and anxiety); (2) History of brain injury or brain surgery; (3) Use of medications or alcohol that could affect brain function (within at least 48.00 h prior to the experiment); (4) Severe myopia (exceeding −6.00D), which may interfere with task performance (Stern and Barulli, 2019). Color blindness or color weakness, which could affect the ability to perform color-related tasks.

Before the experiment began, the purpose and procedures were explained to all participants, and written informed consent was obtained. The study was approved by the Ethics Committee of Guangxi Normal University (Approval Number: 20231226001), and all methods were conducted in accordance with the latest guidelines and regulations of the Declaration of Helsinki.

### 2.3 Experimental procedures

This study aimed to investigate the effects of an 18-week street dance intervention on prefrontal cortex brain function. Functional near-infrared spectroscopy (fNIRS) was used to measure changes in prefrontal oxygenated hemoglobin (HbO₂) levels during three cognitive paradigms: the 2-back task, Stroop task, and More-Odd Shifting task. The experiment was divided into three phases: pre-intervention testing, the intervention phase, and post-intervention testing.

During the pre-intervention testing phase, participants entered the laboratory and acclimated for 15 min in a quiet environment to reduce stress and external interference. Testing sessions were scheduled based on participants’ menstrual cycles to minimize physiological variability. Participants then completed the cognitive tasks (2-back, Stroop, and Shifting), which were presented in randomized order to control for sequence effects. A 19-channel fNIRS system was used to record HbO₂ changes in the prefrontal cortex. Experimenters ensured good probe–scalp contact and monitored signal quality in real time, excluding noisy or invalid channels.

The intervention phase lasted 18 weeks. Participants in the street dance group attended structured street dance sessions three times per week, each lasting 80 min. The program content and progression are detailed in Section 2.4.1. Participants in the control group did not receive any structured exercise or dance training but were instructed to maintain their usual daily routines. Additional details regarding the control condition are described in Section 2.4.2.

**Figure 1:**
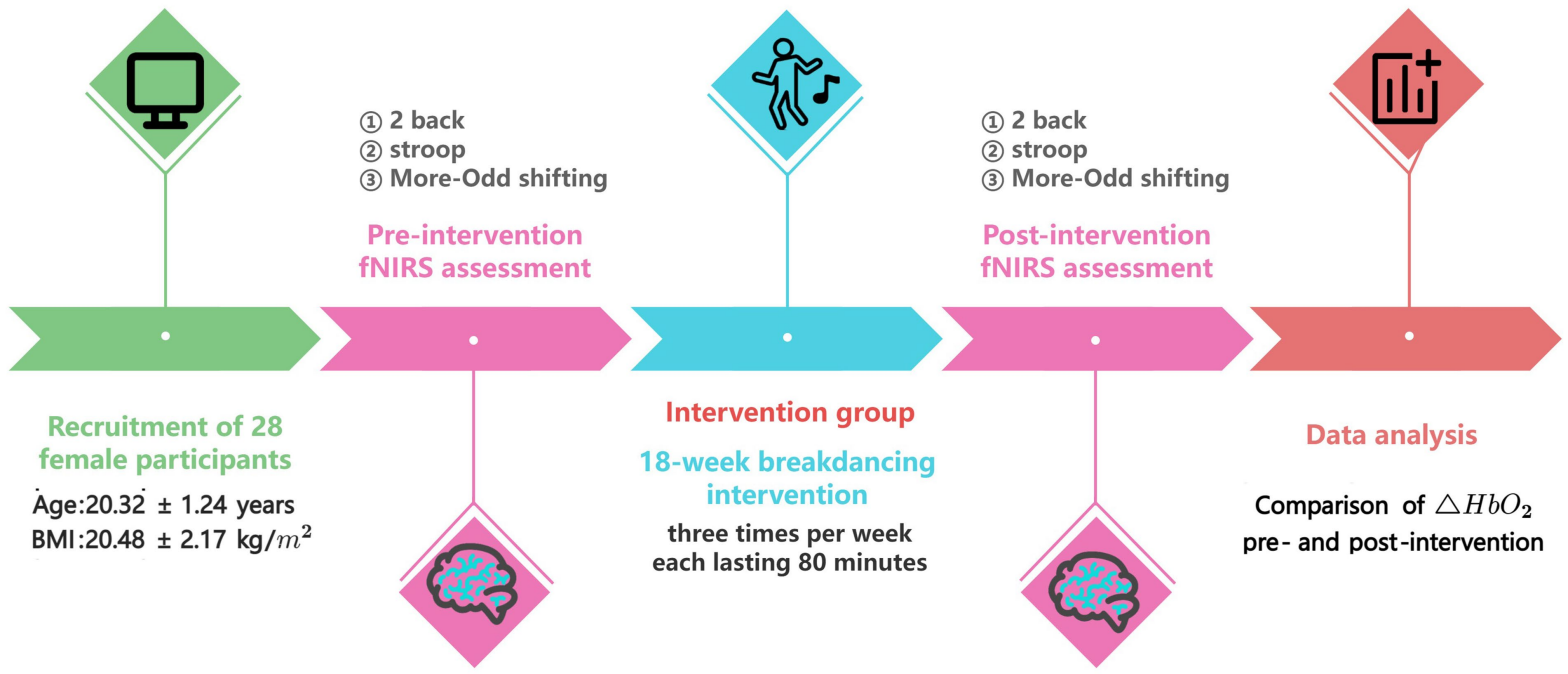
Experimental procedure chart (*n* = 28). △HbO₂, change in oxygenated hemoglobin concentration.

In the post-intervention testing phase, all participants completed the same set of cognitive tasks as in the pre-intervention phase, under identical environmental and equipment conditions. Testing sessions were again arranged to avoid participants’ menstrual periods. The collected fNIRS data were processed to analyze HbO₂ changes across prefrontal cortex channels. Data quality control was performed by excluding noisy or anomalous channels. This methodological rigor ensured robust evaluation of the impact of the street dance intervention compared with the control condition (Figure 1).

### 2.4 Intervention protocol

In this study, street dance specifically refers to dance styles such as Breaking, Locking, and Popping, which are characterized by improvisation, strong rhythmicity, coordinated movement patterns, and complex choreographic sequences. We selected street dance as the intervention because its rapid rhythm changes, demanding spatial transitions, and memory of complex movement sequences impose high cognitive loads on attention, working memory, and executive control. Compared with traditional forms of exercise, street dance therefore provides a more cognitively engaging activity that has the potential to strongly stimulate prefrontal activation and enhance executive function, making it a promising strategy for building cognitive reserve.

#### 2.4.1 Street dance group

In this study, the street dance intervention incorporated commonly practiced styles such as Breaking, Locking, and Popping. These styles are characterized by strong rhythmicity, coordinated movement patterns, and improvisational elements. Street dance was selected as the intervention modality because its complex choreographic sequences and rapid spatial transitions impose high cognitive demands on working memory, attention, and executive control, thereby providing an effective stimulus for prefrontal activation.

The street dance intervention lasted for 18 weeks, with participants attending three sessions per week, each lasting 80 min. All sessions were led by a professional instructor with extensive teaching experience, ensuring a systematic and progressive structure. The program included the following components:

(1) Warm-up and Stretching (15 min): Joint mobility exercises, dynamic stretching, and light aerobic activity, focusing on activating major muscle groups and preparing the body for training.

(2) Basic Movement Practice (40 min):

Popping: Training “Hit” (quick contractions) and “Wave” (wave-like motions) to enhance body control and rhythm.

Locking: Practicing “Pointing” and “Wrist roll,” emphasizing precision and rhythm synchronization.

Groove: Including “Bouncing” and body sways to align with musical rhythm and improve fluidity.

Toprock: Starting with “Side step” and “Cross step,” progressing to more complex footwork to enhance spatial awareness and movement memory.

(3) Dance Combination Learning (20 min): Linking basic movements into choreographed sequences, training flow, rhythm control, and coordination.

(4) Cool-down and Recovery (5 min): Static stretching and breathing exercises to relax muscles and reduce fatigue.

Throughout the intervention, heart rate monitoring ensured that exercise intensity was maintained at 60–75% of maximum heart rate (calculated by the Karvonen formula: HRmax = 220 – age). Participants were required to maintain at least 80% attendance, with noncompliant individuals excluded from final data analysis.

#### 2.4.2 Control group

The control group did not receive any form of dance or structured exercise intervention and was instructed to maintain their regular study and daily life routines as a reference condition. Participants were asked to keep their original daily activities throughout the 18-week study period without engaging in additional structured exercise training or dance classes, performing only light daily physical activity such as walking or commuting.

To ensure compliance and control for potential confounding factors, the research team maintained weekly contact with participants in the control group via phone or online communication. This process confirmed that they had not participated in any additional exercise interventions. If a participant was found to have failed to comply with these requirements, their data were excluded from the analysis.

### 2.5 Blinding and bias control

To minimize experimenter bias, different researchers were assigned to distinct roles during the study. One team of instructors supervised the intervention sessions, while another team of experimenters, who were blinded to group assignment, conducted the pre- and post-intervention cognitive testing and fNIRS data collection. Data analysts were also blinded to participant group allocation during statistical analysis, with datasets coded by ID numbers only. Additionally, standardized testing procedures and pre-defined analysis pipelines were applied to all participants to further reduce subjective bias.

### 2.6 FNIRS data acquisition

**Figure 2:**
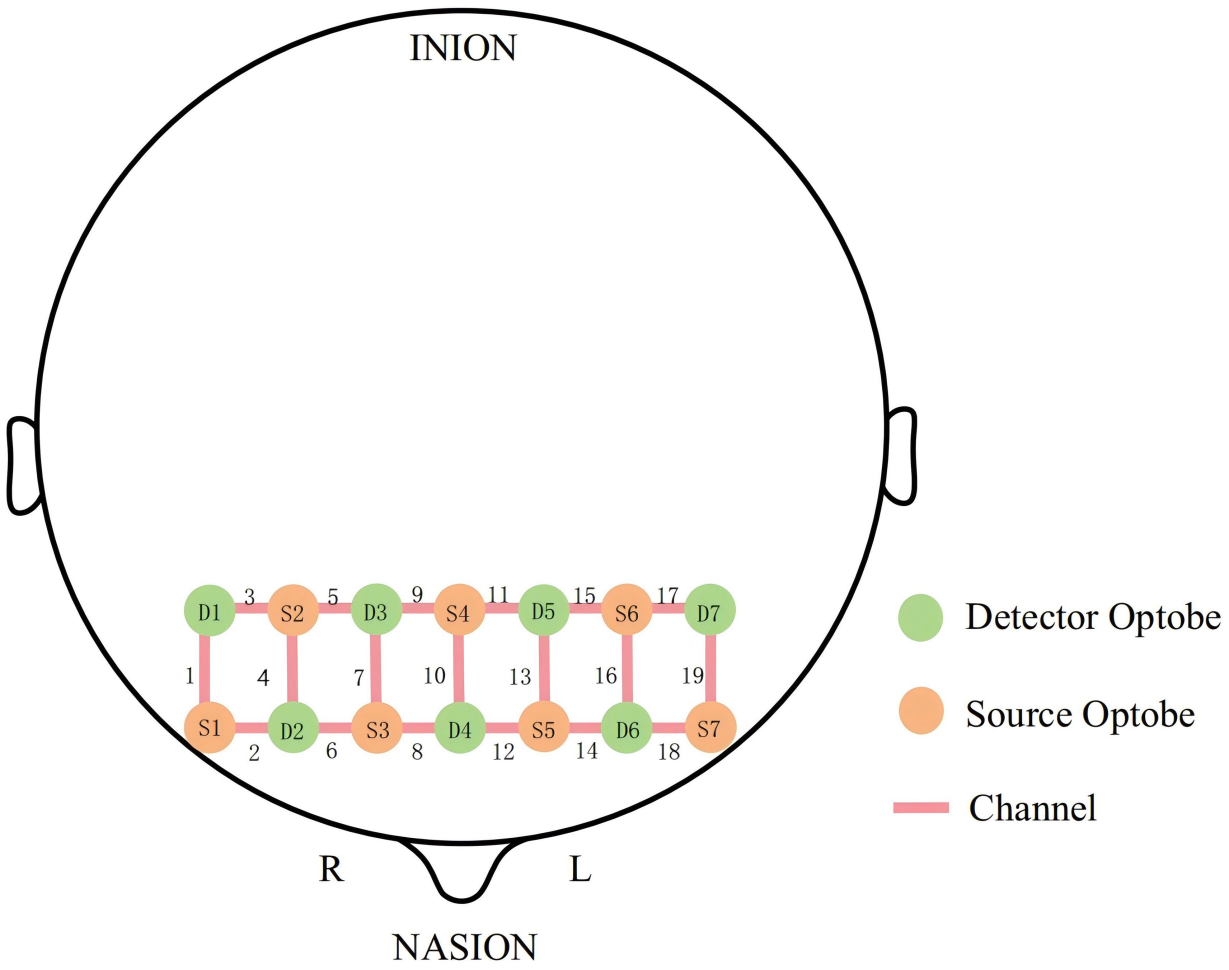
Placement of optode in 19 channels of the prefrontal lobe of the brain based on the international 10/20 system.

This study used a portable functional near-infrared spectroscopy (fNIRS) device (NirSmartII-3000C, Danyang Huichuang Medical Equipment Co., Ltd., China) for signal acquisition. The device utilized light sources with wavelengths of 730 and 850 nm and had a sampling rate of 11 Hz. Figure 2 illustrates the distribution of 19 measurement channels, which were formed by 7 source probes and 7 detector probes. These probes were placed on the participants’ prefrontal cortex following the International 10–20 system standard.

Based on the specific locations of the channels in the prefrontal cortex, they were grouped into six regions of interest (ROI): the right inferior frontal gyrus (RIFG), left inferior frontal gyrus (LIFG), right dorsolateral prefrontal cortex (RDLPFC), left dorsolateral prefrontal cortex (LDLPFC), right frontopolar area (RFPA), and left frontopolar area (LFPA) (Zohdi et al., 2024) (Table 1).

**Table 1:** ROI corresponding to each channel.

ROI	Channels (CH)
Right inferior prefrontal gyrus (RIFG)	CH1, CH2
Left inferior prefrontal gyrus (LIFG)	CH18, CH19
Right dorsolateral prefrontal cortex (RDLPFC)	CH3, CH4, CH5, CH6, CH7
Left dorsolateral prefrontal cortex (LDLPFC)	CH13, CH14, CH15, CH16, CH17
Right frontopolar area (RFPA)	CH8, CH9
Left frontopolar area (LFPA)	CH11, CH12

### 2.7 Experimental paradigm

**Figure 3:**
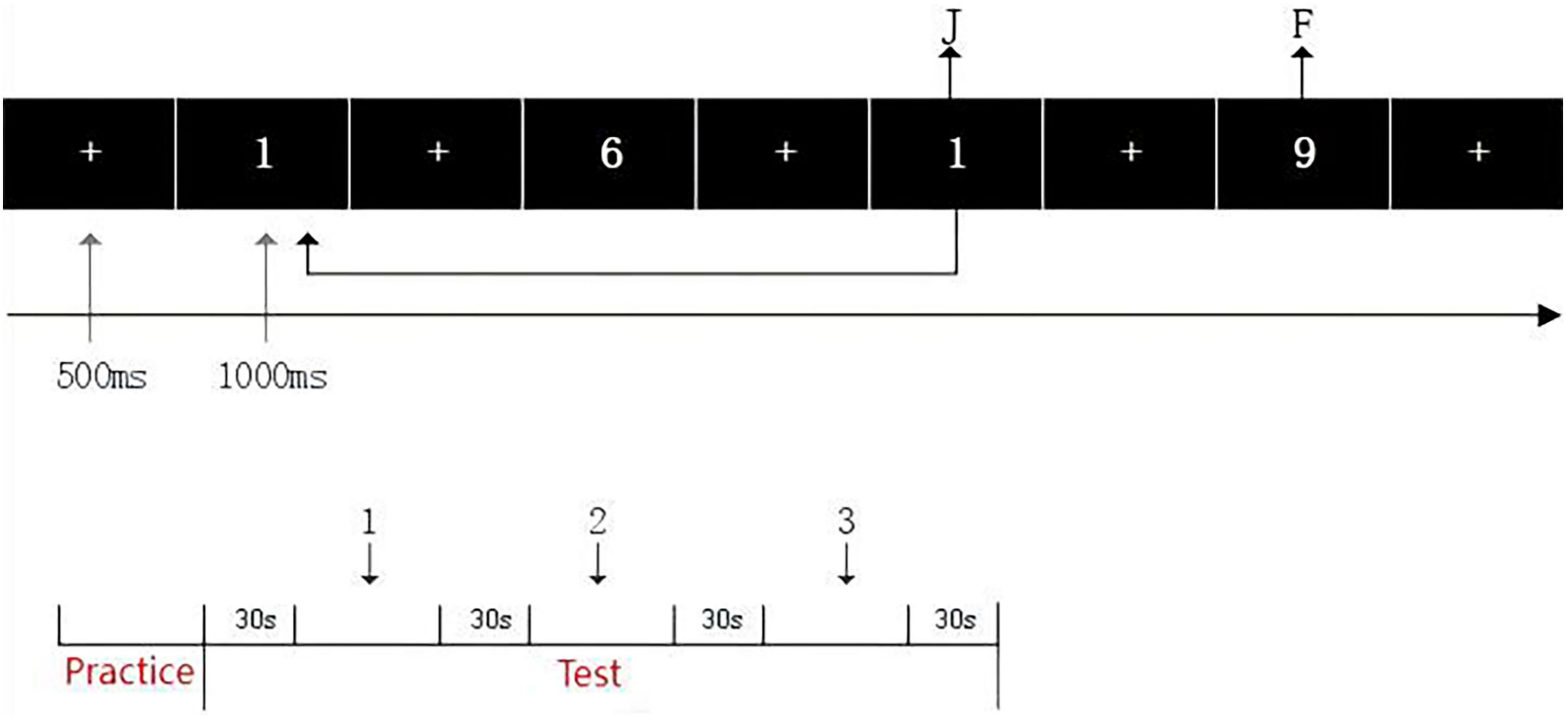
Schematic of the 2-back task procedure.

**Figure 4:**
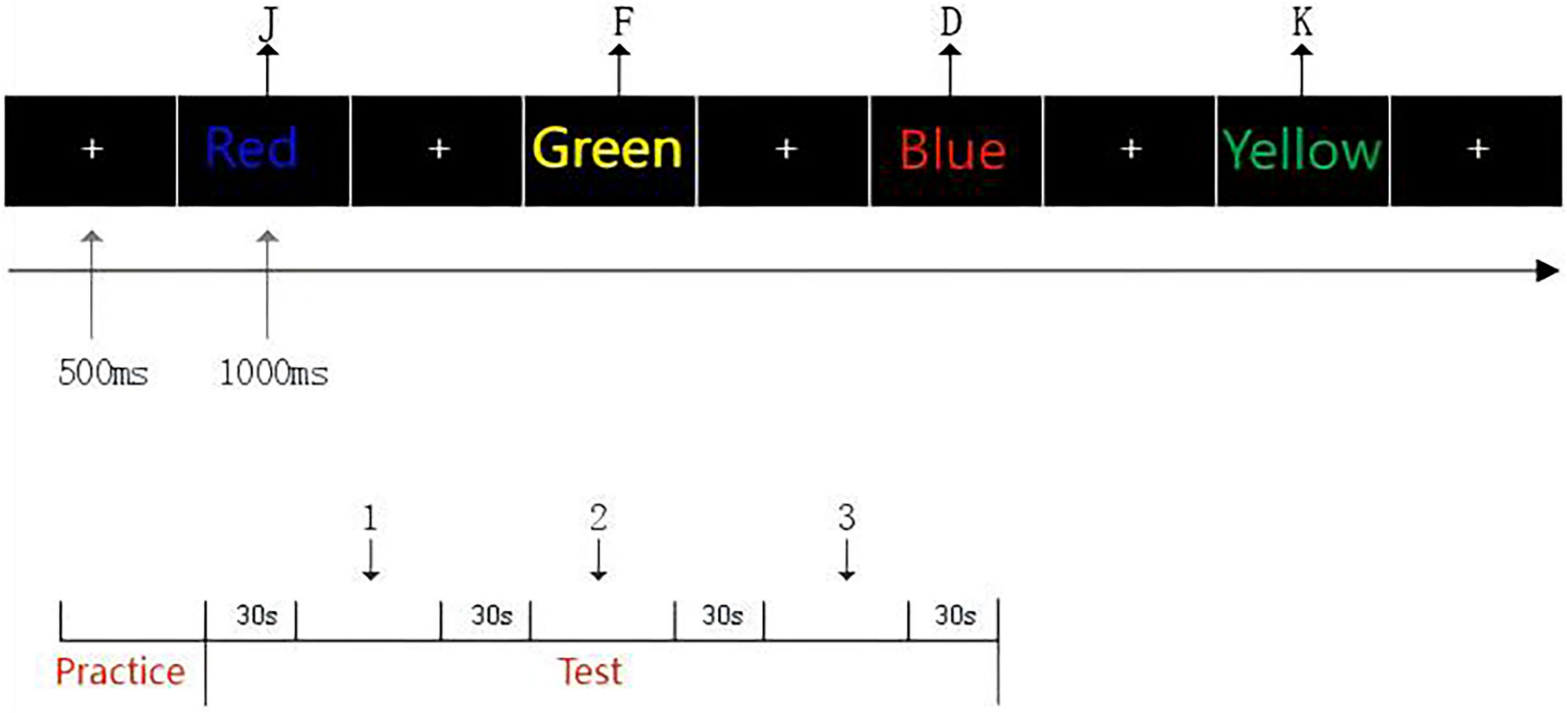
Schematic of the Stroop task procedure.

**Figure 5:**
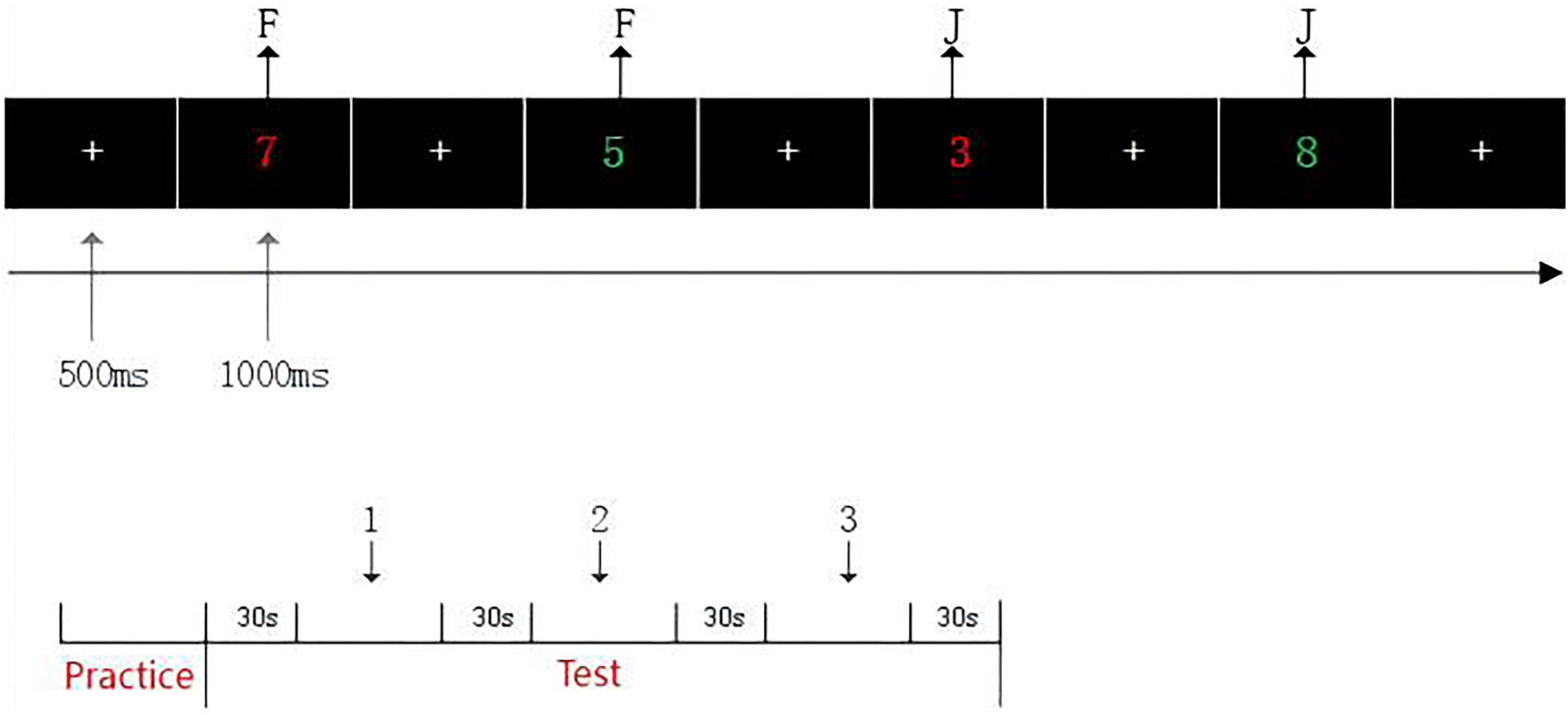
Schematic of the More-Odd Shifting task procedure.

To evaluate the effects of the intervention on cognitive function, three task paradigms were designed to assess inhibitory control, updating function, and shifting function. All tasks were presented using E-Prime software, with each task consisting of three blocks of 48 trials, for a total of 144 trials per task. Each trial included a fixation point (“+”) displayed for 500 milliseconds, followed by a stimulus image presented for 1,000 milliseconds. A 30-s rest period was provided between blocks to minimize fatigue. The specific task designs are described as follows (Figures 3–5).

### 2.8 fNIRS data preprocessing

The fNIRS data were preprocessed using NirSpark software, following these steps: (1) Channels with high noise due to poor probe contact or severe motion artifacts were excluded, with contaminated segments corrected by spline interpolation or removed if necessary. Motion artifacts were identified through both visual inspection and automated detection in NirSpark. (2) The light intensity signals were converted into optical density signals. (3) A bandpass filter (0.01–0.2 Hz) was applied to remove low-frequency drifts and high-frequency noise. (4) The optical density data were converted into concentration changes of oxygenated hemoglobin (HbO₂) and deoxygenated hemoglobin (HbR) using the modified Beer–Lambert law. (5) The hemodynamic response function (HRF) was set to start at −2 s and end at 102 s, with the baseline defined as −2 to 0 s. The time window for a single task block was defined as 0–102 s. Task-related oxygenation signals were then averaged across trials to generate block-averaged results for subsequent analysis (Wang et al., 2020; Li et al., 2020).

For statistical analyses, preprocessing was performed at the channel level, but main results were summarized at the ROI level (RDLPFC, LDLPFC, RFPA, LFPA, RIFG, LIFG), based on prior evidence linking these regions to working memory, inhibitory control, and cognitive flexibility.

### 2.9 Statistical analysis

All statistical analyses were conducted using SPSS 26.0 (Chicago, IL, United States). Data are presented as mean ± standard deviation (SD). To evaluate the effects of the 18-week street dance intervention on prefrontal activation, a two-way repeated-measures analysis of variance (ANOVA) was performed, with Group (Street dance vs. Control) as the between-subjects factor and Time (Pre- vs. Post-intervention) as the within-subjects factor. Separate ANOVAs were conducted for the three tasks (2-back, Stroop, and More-Odd Shifting).

When significant Group × Time interactions were observed, simple effects analyses were performed. In addition, paired-samples *t*-tests were conducted within each group to provide a more intuitive comparison of pre- and post-intervention changes.

Effect sizes (Cohen’s *d*) were calculated and interpreted according to Cohen’s criteria: small (*d* = 0.2), medium (*d* = 0.5), and large (*d* = 0.8) (Lakens, 2013).

To account for the increased risk of Type I error due to multiple channel and task comparisons, *p*-values were adjusted using the Benjamini–Hochberg False Discovery Rate (FDR) correction with *q* = 0.05 applied within each task. All reported significant results are based on FDR-adjusted values unless otherwise noted as exploratory.

## 3 Results

Baseline characteristics are presented in Table 2. No significant between-group differences were observed in age or BMI (all *p* > 0.05). All participants were right-handed, had normal or corrected-to-normal vision, and reported no history of neurological or psychiatric disorders, supporting the comparability of the two groups at baseline.

**Table 2:** Baseline characteristics of participants in the Street Dance and Control groups.

Characteristic	Street Dance (*n* = 14)	Control (*n* = 14)	*p*-value
Age (years)	20.07 ± 0.87	20.15 ± 1.41	0.873
BMI (kg/m^2^)	19.82 ± 1.91	20.41 ± 2.16	0.453

### 3.1 HbO₂ changes in the 2-back task pre- and post-intervention

**Figure 6:**
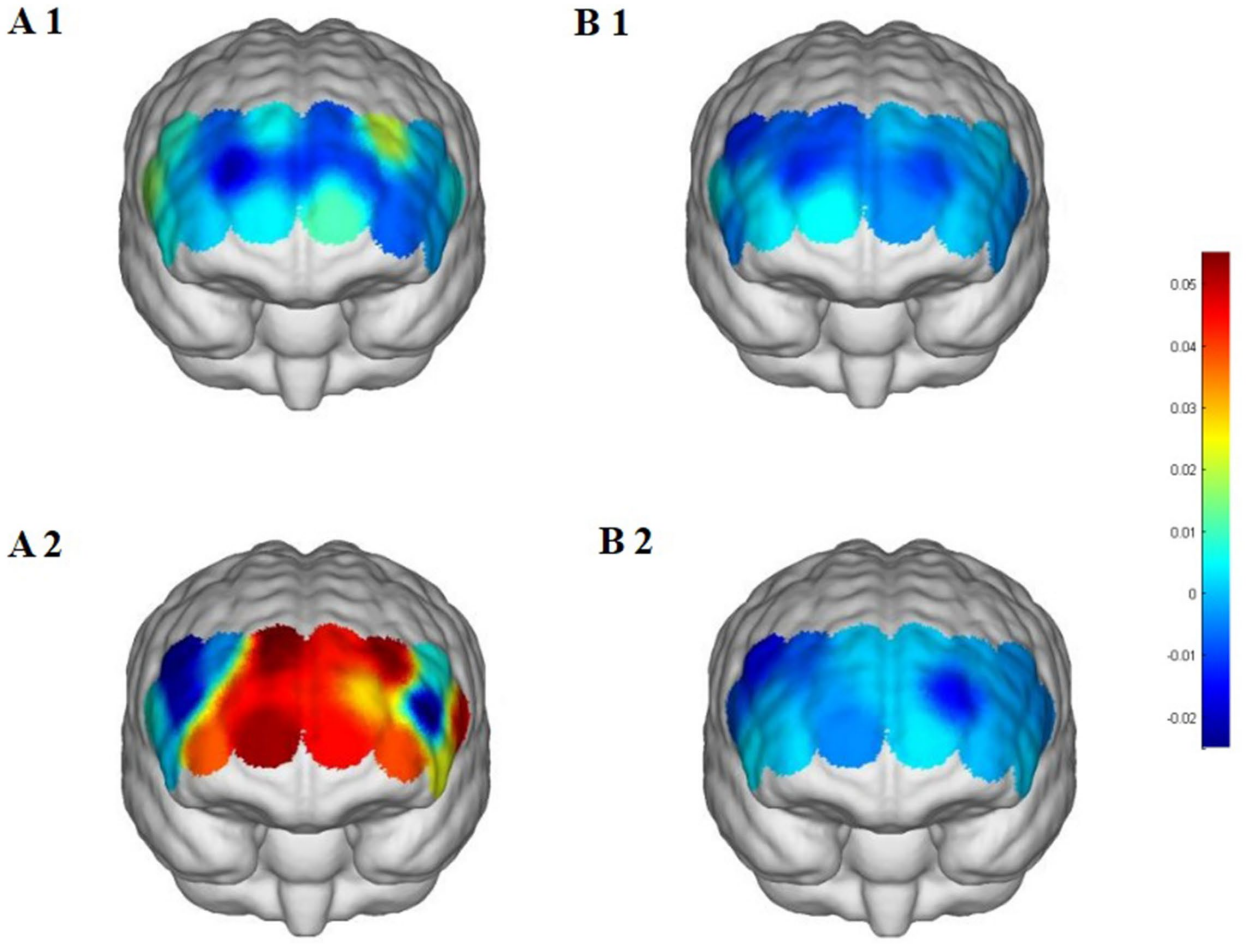
Prefrontal cortex △HbO₂ changes during the 2-back task. **(A1,A2)** Street dance group pre/post; **(B1,B2)** control group pre/post. △HbO₂, change in oxygenated hemoglobin concentration.

Two-way repeated-measures ANOVA revealed significant or marginally significant Group × Time interaction effects across several prefrontal regions. Specifically, the street dance group exhibited significant HbO₂ increases in the right dorsolateral prefrontal cortex (CH7, CH10), right frontopolar area (CH8, CH9), left dorsolateral prefrontal cortex (CH13, CH14, CH15), and the left inferior frontal gyrus (CH19), suggesting enhanced working memory load processing, cognitive control, and semantic-related functions. Additional increases were also observed in the left frontopolar area (CH11) and left inferior frontal gyrus (CH18) (Table 3 and Figure 6).

**Table 3:** Prefrontal cortex △HbO₂ changes during the 2-back task pre- and post-intervention (×10^−2^ mmol/L, Mean ± SD).

Channel	Group	Pre-intervention	Post-intervention	*t*-value	*p*-value	Cohen’s *d* (95% CI)
CH1	Street dance group	1.53 ± 2.56	0.46 ± 7.96	−0.51	0.620	0.18 (−0.40, 0.76)
	Control group	0.50 ± 3.00	−0.80 ± 3.10	−1.10	0.295	0.25 (−0.34, 0.84)
CH2	Street dance group	0.33 ± 2.55	−0.17 ± 6.59	−0.22	0.829	0.10 (−0.48, 0.68)
	Control group	−0.50 ± 2.80	0.30 ± 2.90	0.92	0.367	0.15 (−0.43, 0.73)
CH3	Street dance group	0.61 ± 3.02	−2.60 ± 3.83*	−2.38	0.034	0.93 (0.23, 1.63)
	Control group	−1.20 ± 2.50	−1.50 ± 2.55	−0.50	0.620	0.12 (−0.46, 0.70)
CH4	Street dance group	−0.33 ± 3.04	−2.24 ± 4.34	−1.06	0.306	0.51 (−0.11, 1.13)
	Control group	0.10 ± 2.40	−0.10 ± 2.50	−0.40	0.710	0.08 (−0.50, 0.66)
CH5	Street dance group	−1.02 ± 2.01	−0.64 ± 3.80	0.30	0.770	0.13 (−0.45, 0.71)
	Control group	−0.70 ± 2.00	−0.80 ± 2.10	−0.50	0.630	0.10 (−0.48, 0.68)
CH6	Street dance group	−0.16 ± 3.82	3.79 ± 5.72	1.61	0.132	0.81 (0.14, 1.48)
	Control group	0.30 ± 2.90	0.10 ± 3.10	−0.60	0.550	0.10 (−0.48, 0.68)
CH7	Street dance group	−2.38 ± 3.47	4.36 ± 5.13**	3.16	0.007	1.54 (0.67, 2.41)
	Control group	−1.20 ± 3.30	−0.30 ± 3.40	0.90	0.370	0.12 (−0.46, 0.70)
CH8	Street dance group	0.03 ± 3.12	5.40 ± 4.55**	3.14	0.008	1.38 (0.56, 2.20)
	Control group	0.50 ± 2.50	−0.50 ± 3.10	−1.00	0.315	0.25 (−0.34, 0.84)
CH9	Street dance group	0.29 ± 2.41	5.58 ± 5.41**	3.43	0.004	1.26 (0.47, 2.05)
	Control group	−0.90 ± 2.60	0.10 ± 2.80	1.10	0.270	0.15 (−0.43, 0.73)
CH10	Street dance group	−1.48 ± 2.67	4.32 ± 4.05***	4.13	<0.001	1.69 (0.77, 2.61)
	Control group	−0.60 ± 2.30	0.10 ± 2.50	0.70	0.485	0.08 (−0.50, 0.66)
CH11	Street dance group	−1.20 ± 4.10	4.36 ± 5.86*	2.45	0.029	1.10 (0.36, 1.84)
	Control group	0.01 ± 2.00	0.10 ± 2.30	0.30	0.740	0.05 (−0.53, 0.63)
CH12	Street dance group	0.89 ± 4.63	4.46 ± 5.91	1.51	0.155	0.67 (0.03, 1.31)
	Control group	−0.40 ± 2.90	0.20 ± 3.10	0.60	0.540	0.15 (−0.43, 0.73)
CH13	Street dance group	−1.33 ± 4.81	2.70 ± 4.11*	2.81	0.015	0.90 (0.21, 1.59)
	Control group	−1.00 ± 3.50	−1.50 ± 3.80	−0.70	0.490	0.15 (−0.43, 0.73)
CH14	Street dance group	−1.08 ± 2.78	3.93 ± 5.31*	2.40	0.032	1.18 (0.42, 1.94)
	Control group	0.01 ± 2.10	−0.20 ± 2.50	−0.60	0.550	0.10 (−0.48, 0.68)
CH15	Street dance group	1.88 ± 2.38	5.31 ± 2.98***	4.59	<0.001	1.27 (0.48, 2.06)
	Control group	−0.20 ± 2.50	0.10 ± 2.60	0.60	0.550	0.10 (−0.48, 0.68)
CH16	Street dance group	−0.78 ± 2.18	−2.90 ± 6.87	−0.97	0.348	0.42 (−0.18, 1.02)
	Control group	0.10 ± 2.40	−0.30 ± 2.50	−0.50	0.625	0.12 (−0.46, 0.70)
CH17	Street dance group	−0.31 ± 2.57	0.40 ± 3.43	0.48	0.639	0.23 (−0.36, 0.82)
	Control group	0.20 ± 2.10	−0.40 ± 2.30	−1.50	0.070	0.25 (−0.34, 0.84)
CH18	Street dance group	−0.46 ± 3.30	2.19 ± 5.49*	2.73	0.017	0.58 (−0.05, 1.21)
	Control group	0.30 ± 2.50	0.10 ± 2.60*	−1.00	0.025	0.40 (−0.20, 1.00)
CH19	Street dance group	0.28 ± 2.91	4.52 ± 7.56*	2.69	0.019	0.74 (0.08, 1.40)
	Control group	−0.10 ± 2.30	−0.20 ± 2.40*	−1.50	0.043	0.28 (−0.31, 0.87)

*p < 0.05, **p < 0.01, ***p < 0.001.

Apart from these findings, most other channels did not exhibit significant changes, indicating regional specificity of the intervention effects. The control group, which maintained their usual daily activities, showed no comparable changes, further confirming that the observed HbO₂ improvements were attributable to the street dance intervention rather than non-specific or repeated testing effects.

At baseline, no significant group differences were observed in accuracy [*F*(1,26) = 1.08, *p* = 0.31] or reaction time (*F* = 0.91, *p* = 0.410).

After the intervention, the street dance group showed a significant improvement in accuracy (+6.09%, 84.80% → 90.89%; *t* = 3.36, *p* = 0.003, *d* = 0.72) and a significant reduction in reaction time (−109.53 ms, 801.42 ± 103.62 → 691.89 ± 171.15 ms; *t* = −4.31, *p* = 0.001, *d* = 1.24), indicating enhanced working memory processing efficiency.

In contrast, the control group exhibited a non-significant decrease in accuracy (−2.62%, 82.39% → 79.43%; *t* = −1.61, *p* = 0.122, *d* = 0.61) and a non-significant reduction in reaction time (−45.89 ms, 804.34 ± 132.48 → 758.63 ± 145.43 ms; *t* = −0.66, *p* = 0.513, *d* = 0.28).

### 3.2 HbO₂ changes in the Stroop task pre- and post-intervention

**Figure 7:**
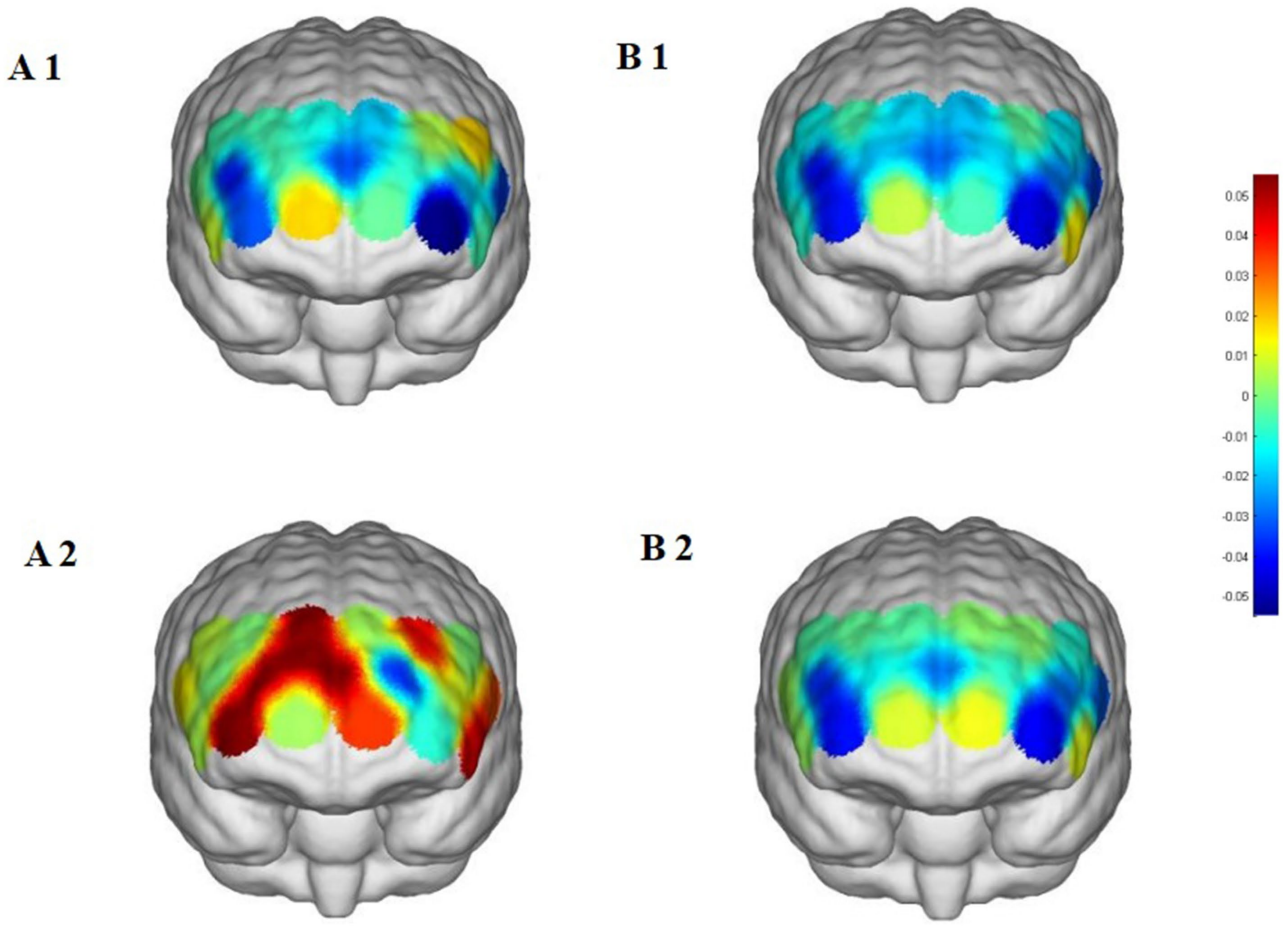
Prefrontal cortex △HbO₂ changes during the stroop task. **(A1,A2)** Street dance group pre/post; **(B1,B2)** Control group pre/post.

Two-way repeated-measures ANOVA revealed significant or marginally significant Group × Time interaction effects across several prefrontal regions. In the street dance group, HbO₂ levels significantly increased in the right dorsolateral prefrontal cortex (CH6, CH7), the right frontopolar area (CH9), and the right dorsolateral prefrontal cortex (CH10), suggesting enhanced task rule maintenance, conflict monitoring, and inhibitory control. Moreover, significant increases were also observed in the left dorsolateral prefrontal cortex (CH15, CH14) and the left inferior frontal gyrus (CH18, CH19), reflecting improvements in executive control, semantic processing, and task switching (Table 4 and Figure 7).

**Table 4:** Prefrontal cortex △HbO₂ changes during the Stroop task pre- and post-intervention (×10^−2^ mmol/L, Mean ± SD).

Channel	Group	Pre-Intervention	Post-Intervention	*t*-value	*p*-value	Cohen’s *d* (95% CI)
CH1	Street dance group	−0.55 ± 5.69	1.16 ± 4.06	0.82	0.427	0.35 (−0.52, 1.22)
	Control group	−1.45 ± 4.10	0.32 ± 3.50	1.20	0.245	0.28 (−0.37, 0.93)
CH2	Street dance group	0.42 ± 6.99	0.12 ± 6.46	−0.15	0.887	0.04 (−0.68, 0.76)
	Control group	−0.92 ± 3.18	0.25 ± 2.95	1.15	0.290	0.30 (−0.33, 0.93)
CH3	Street dance group	−0.98 ± 5.16	0.30 ± 6.51	0.58	0.572	0.22 (−0.56, 1.00)
	Control group	−1.33 ± 3.60	−0.48 ± 3.20	1.25	0.210	0.33 (−0.26, 0.92)
CH4	Street dance group	−4.24 ± 6.15	−0.39 ± 7.16	1.85	0.087	0.58 (−0.09, 1.25)
	Control group	−4.02 ± 5.10	−3.67 ± 4.20	0.90	0.370	0.25 (−0.30, 0.80)
CH5	Street dance group	−0.96 ± 4.40	−0.32 ± 4.80	0.38	0.708	0.14 (−0.61, 0.89)
	Control group	−0.58 ± 3.10	−0.31 ± 2.90	0.72	0.475	0.20 (−0.36, 0.76)
CH6	Street dance group	−3.57 ± 8.12	5.12 ± 8.14**	3.53	0.004	1.07 (0.33, 1.81)
	Control group	−3.98 ± 4.20	−4.01 ± 4.30	−0.10	0.920	0.01 (−0.54, 0.56)
CH7	Street dance group	−0.95 ± 13.81	4.99 ± 7.36	1.91	0.078	0.54 (−0.07, 1.15)
	Control group	−2.05 ± 4.60	−1.35 ± 4.05	1.35	0.190	0.40 (−0.20, 1.00)
CH8	Street dance group	1.53 ± 8.81	−0.03 ± 5.65	−0.59	0.565	0.21 (−0.57, 0.99)
	Control group	0.62 ± 3.12	0.85 ± 3.20	1.05	0.310	0.22 (−0.32, 0.76)
CH9	Street dance group	−1.49 ± 7.26	4.94 ± 6.33**	3.07	0.009	0.94 (0.25, 1.63)
	Control group	−1.75 ± 3.55	−0.45 ± 3.18	1.37	0.190	0.37 (−0.23, 0.97)
CH10	Street dance group	−3.62 ± 10.99	5.09 ± 5.19**	3.13	0.008	1.01 (0.29, 1.73)
	Control group	−3.02 ± 4.50	−2.78 ± 4.30	0.80	0.430	0.25 (−0.32, 0.82)
CH11	Street dance group	−2.25 ± 3.96	0.02 ± 6.71	1.27	0.227	0.41 (−0.26, 1.08)
	Control group	−1.98 ± 4.10	0.14 ± 3.12*	2.70	0.016	0.65 (0.13, 1.17)
CH12	Street dance group	−0.64 ± 12.20	3.20 ± 7.43	1.24	0.235	0.38 (−0.31, 1.07)
	Control group	−0.58 ± 3.20	1.05 ± 3.25	1.50	0.145	0.40 (−0.14, 0.94)
CH13	Street dance group	−1.24 ± 5.57	−3.95 ± 5.67	−1.20	0.253	0.48 (−0.24, 1.20)
	Control group	−1.80 ± 3.70	−0.90 ± 3.50*	2.06	0.038	0.49 (0.03, 0.95)
CH14	Street dance group	−5.81 ± 3.72	−1.27 ± 9.10	1.81	0.093	0.65 (−0.06, 1.36)
	Control group	−4.56 ± 5.30	−4.32 ± 5.10	0.38	0.700	0.05 (−0.49, 0.59)
CH15	Street dance group	0.17 ± 3.72	3.65 ± 5.01*	2.46	0.029	0.79 (0.09, 1.49)
	Control group	−0.35 ± 2.85	0.12 ± 3.10	0.80	0.420	0.16 (−0.38, 0.70)
CH16	Street dance group	−2.25 ± 5.46	0.68 ± 6.24	1.54	0.149	0.50 (−0.18, 1.18)
	Control group	−2.74 ± 4.80	−2.88 ± 4.90	−0.35	0.740	0.10 (−0.45, 0.65)
CH17	Street dance group	1.42 ± 7.52	−0.32 ± 6.68	−0.72	0.482	0.24 (−0.50, 0.98)
	Control group	−1.55 ± 3.85	−1.00 ± 3.63*	1.25	0.045	0.50 (0.01, 0.99)
CH18	Street dance group	−0.95 ± 7.03	4.10 ± 4.90*	2.99	0.010	0.83 (0.21, 1.45)
	Control group	1.50 ± 3.60	1.10 ± 3.20	−0.60	0.530	0.22 (−0.32, 0.76)
CH19	Street dance group	−3.74 ± 6.69	2.82 ± 3.39**	3.04	0.009	1.24 (0.39, 2.09)
	Control group	−3.41 ± 4.20	−3.08 ± 4.30	0.60	0.555	0.10 (−0.45, 0.65)

*p < 0.05, **p < 0.01.

Beyond these findings, most channels did not show significant changes, highlighting the regional specificity of the intervention effects. No comparable increases were observed in the control group, which maintained daily activities, further supporting that the observed HbO₂ improvements in the Stroop task were attributable to the street dance intervention.

At baseline, no significant group differences were found in overall accuracy (*F* = 0.79, *p* = 0.460), reaction time (*F* = 1.77, *p* = 0.178), accuracy under the incongruent condition (*F* = 2.21, *p* = 0.118), or reaction time under the incongruent condition (*F* = 0.66, *p* = 0.520).

In the congruent condition, the street dance group showed a significant improvement in accuracy (+2.85%, *t* = 3.47, *p* = 0.002, *d* = 0.76), while the control group showed a significant decrease (−2.06%, *t* = −2.18, *p* = 0.041, *d* = 0.48). Reaction times decreased significantly in the street dance group (−50.96 ms, *t* = −3.03, *p* = 0.007, *d* = 0.66), whereas the control group showed no significant change (−5.99 ms, *p* = 0.798).

In the incongruent condition, the street dance group exhibited a significant improvement in accuracy (+4.25%, *t* = 4.82, *p* < 0.001, *d* = 1.05), while the control group showed a small, non-significant increase (+1.70%, *p* = 0.972). Reaction times in the street dance group decreased significantly (−49.58 ms, *t* = −3.03, *p* = 0.007, *d* = 0.66), whereas the control group showed no significant change (−3.53 ms, *p* = 0.513).

### 3.3 HbO₂ changes in the more-odd shifting task pre- and post-intervention

**Figure 8:**
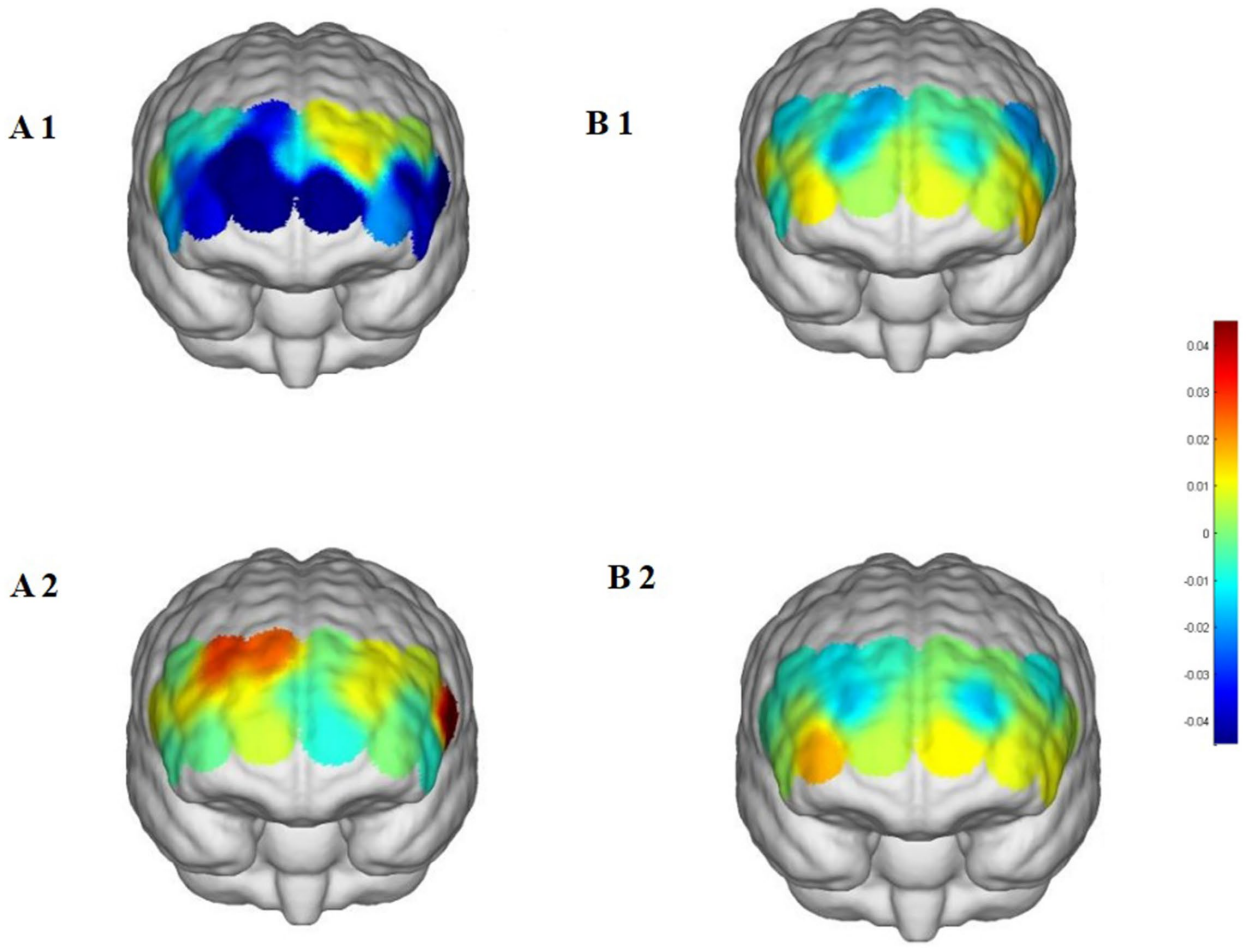
Prefrontal cortex △HbO₂ changes during the More-Odd Shifting task. **(A1,A2)** Street dance group pre/post; **(B1,B2)** control group pre/post.

Two-way repeated-measures ANOVA revealed significant or marginally significant Group × Time interaction effects across several prefrontal regions. In the street dance group, HbO₂ significantly increased in the right dorsolateral prefrontal cortex (CH4, CH5, and CH7), suggesting enhanced cognitive control and rule updating. The right frontopolar area (CH8 and CH9) also showed significant or marginal increases, reflecting improvements in attentional reallocation and cognitive flexibility. Additionally, the left frontopolar area (CH12) exhibited a trend toward increased activation, while the left inferior frontal gyrus (CH19) demonstrated a robust increase, indicating enhanced semantic processing and inhibitory functions (Table 5 and Figure 8).

**Table 5:** Prefrontal cortex △HbO₂ changes during the More-Odd Shifting task pre- and post-intervention (×10^−2^ mmol/L, Mean ± SD).

Channel	Group	Pre-Intervention	Post-Intervention	*t*-value	*p*-value	Cohen’s *d* (95% CI)
CH1	Street dance group	0.52 ± 7.30	0.97 ± 7.58	0.18	0.862	0.06 (−0.46, 0.58)
	Control group	1.20 ± 4.30	−0.50 ± 3.10	−1.50	0.145	0.30 (−0.24, 0.84)
CH2	Street dance group	−1.89 ± 7.69	−0.41 ± 7.85	0.58	0.575	0.19 (−0.34, 0.72)
	Control group	−0.90 ± 3.10	0.30 ± 2.80	1.20	0.245	0.25 (−0.28, 0.78)
CH3	Street dance group	−0.79 ± 5.47	−0.12 ± 5.31	0.34	0.738	0.12 (−0.41, 0.65)
	Control group	−1.30 ± 2.50	−0.80 ± 3.00	0.60	0.530	0.18 (−0.35, 0.71)
CH4	Street dance group	−2.81 ± 6.62	1.20 ± 6.00*	2.27	0.041	0.63 (0.06, 1.20)
	Control group	0.60 ± 3.00	0.40 ± 2.50	−0.50	0.630	0.10 (−0.43, 0.63)
CH5	Street dance group	−0.65 ± 4.61	2.81 ± 2.48*	2.56	0.024	0.94 (0.31, 1.57)
	Control group	−0.50 ± 2.70	−1.20 ± 3.40*	−1.20	0.023	0.40 (−0.14, 0.94)
CH6	Street dance group	−3.78 ± 9.87	−0.19 ± 5.96	1.13	0.279	0.44 (−0.11, 0.99)
	Control group	1.20 ± 3.10	1.80 ± 3.20	1.10	0.300	0.35 (−0.19, 0.89)
CH7	Street dance group	−8.11 ± 13.68	1.01 ± 4.82	2.01	0.066	0.89 (0.27, 1.51)
	Control group	−2.00 ± 4.50	−1.50 ± 4.10	0.70	0.480	0.20 (−0.33, 0.73)
CH8	Street dance group	−6.99 ± 12.38	0.62 ± 3.74	1.93	0.076	0.83 (0.22, 1.44)
	Control group	0.30 ± 2.90	0.50 ± 3.00	0.90	0.380	0.10 (−0.43, 0.63)
CH9	Street dance group	−3.35 ± 6.46	2.52 ± 4.91*	2.23	0.044	1.02 (0.37, 1.67)
	Control group	−1.80 ± 3.50	−0.70 ± 2.90	1.30	0.200	0.35 (−0.19, 0.89)
CH10	Street dance group	−1.34 ± 11.91	−0.51 ± 4.30	0.22	0.828	0.09 (−0.43, 0.61)
	Control group	0.50 ± 3.10	0.20 ± 2.50	−0.60	0.550	0.12 (−0.41, 0.65)
CH11	Street dance group	1.01 ± 2.22	−0.01 ± 6.82	−0.64	0.535	0.20 (−0.33, 0.73)
	Control group	−0.30 ± 2.40	0.10 ± 2.80	0.800	0.420	0.20 (−0.33, 0.73)
CH12	Street dance group	−7.67 ± 15.52	−0.82 ± 3.86	1.50	0.158	0.61 (0.04, 1.18)
	Control group	0.80 ± 3.30	1.10 ± 3.60	1.00	0.175	0.30 (−0.24, 0.84)
CH13	Street dance group	1.38 ± 4.43	0.98 ± 6.09	−0.27	0.792	0.07 (−0.45, 0.59)
	Control group	−1.10 ± 3.20	−1.60 ± 2.80*	−1.50	0.023	0.50 (−0.06, 1.06)
CH14	Street dance group	−2.19 ± 4.65	0.01 ± 4.56	1.26	0.230	0.48 (−0.07, 1.03)
	Control group	0.60 ± 2.80	0.90 ± 3.10	1.10	0.280	0.25 (−0.28, 0.78)
CH15	Street dance group	0.85 ± 2.99	1.05 ± 3.36	0.15	0.886	0.06 (−0.46, 0.58)
	Control group	−0.20 ± 3.00	0.10 ± 2.70	0.80	0.420	0.15 (−0.38, 0.68)
CH16	Street dance group	−4.19 ± 10.70	−0.52 ± 6.80	1.00	0.337	0.41 (−0.14, 0.96)
	Control group	1.00 ± 2.90	0.50 ± 2.80	−1.00	0.360	0.30 (−0.24, 0.84)
CH17	Street dance group	0.40 ± 12.46	0.76 ± 1.67	0.10	0.923	0.04 (−0.48, 0.56)
	Control group	−2.00 ± 4.20	−1.00 ± 3.50*	1.20	0.010	0.40 (−0.14, 0.94)
CH18	Street dance group	−3.54 ± 12.22	−0.83 ± 7.26	0.72	0.486	0.27 (−0.26, 0.80)
	Control group	1.50 ± 3.50	1.00 ± 3.20	−0.7	0.48	0.20 (−0.33, 0.73)
CH19	Street dance group	−4.39 ± 4.68	4.54 ± 6.21**	3.81	0.002	1.62 (0.82, 2.42)
	Control group	−1.50 ± 3.60	0.50 ± 2.90	2.00	0.05	0.50 (−0.06, 1.06)

*p < 0.05, **p < 0.01.

Beyond these findings, most other channels showed no significant changes, highlighting the regional specificity of the intervention effects. No comparable increases were observed in the control group, further confirming that the improvements in HbO₂ were attributable to the street dance intervention.

At baseline, no significant group differences were observed in task performance (switch accuracy: *F* = 1.62, *p* = 0.207; switch reaction time: *F* = 1.14, *p* = 0.328; non-switch accuracy: *F* = 0.63, *p* = 0.536; non-switch reaction time: *F* = 1.40, *p* = 0.254).

In the switch condition, the street dance group showed a significant improvement in accuracy (+14.41%, *t* = 8.51, *p* = 0.001, *d* = 2.43), while the control group showed a smaller increase (+3.70%, *t* = 2.81, *p* = 0.011, *d* = 0.77). Reaction times decreased significantly in the street dance group (−109.88 ms, *t* = 4.87, *p* < 0.001, *d* = 1.38), compared to a more modest reduction in the control group (−61.24 ms, *t* = 2.37, *p* = 0.028, *d* = 0.64). These findings suggest that street dance training yielded stronger benefits for task-switching accuracy and processing speed.

In the non-switch condition, the street dance group demonstrated a significant increase in accuracy (+5.06%, *t* = 3.58, *p* = 0.002, *d* = 0.82), while the control group showed a minimal, non-significant improvement (+0.64%, *t* = 0.44, *p* = 0.668, *d* = 0.14). Reaction times also decreased significantly in the street dance group (−77.06 ms, *t* = 4.48, *p* < 0.001, *d* = 1.51), with the control group showing a smaller yet significant reduction (−59.86 ms, *t* = 2.83, *p* = 0.010, *d* = 1.05).

## 4 Discussion

### 4.1 2-back task: enhancements in working memory

In the 2-back task, the street dance intervention significantly increased HbO₂ levels in the right dorsolateral prefrontal cortex (RDLPFC) and the right frontopolar area (RFPA), indicating substantial improvements in participants’ working memory capacity. The dorsolateral prefrontal cortex is a core brain region for working memory, responsible for the dynamic storage and manipulation of information, particularly under high cognitive load where its activation is directly associated with task performance (Miller et al., 2018; Bastos et al., 2018). Additionally, the significant activation of the RFPA reflects enhanced cognitive control capabilities in the face of task complexity. The pronounced activation of the left inferior frontal gyrus (LIFG) may suggest improved semantic memory processing when handling task-relevant information. These findings demonstrate that street dance intervention exerts significant neurophysiological effects on promoting the core component of executive function—working memory (Owen et al., 2005; Barbey et al., 2013).

Consistent with these neural findings, behavioral results further reinforce this conclusion. After the intervention, the street dance group showed a significant improvement in accuracy (+6.09%) and a marked reduction in reaction time (−109.53 ms), while the control group exhibited a slight, non-significant decrease in accuracy and a non-significant reduction in reaction time. This close correspondence between behavioral improvements and neural activation patterns strengthens the ecological validity of the findings, suggesting that street dance not only alters prefrontal cortical activation but also produces tangible benefits in working memory performance.

The high demands of action memory and complex sequence coordination in street dance may represent a key mechanism underlying these improvements. Street dance requires participants to memorize and accurately perform complex sequences of movements, a process that relies heavily on cognitive engagement in addition to physical memory (Karpati et al., 2015). The frequent activation of the RDLPFC likely reflects the cognitive load imposed by these tasks. Rapid shifts in movement, position, and formation further increase demands on short-term memory and attention, potentially enhancing working memory efficiency over time. Moreover, the synchronization of music and rhythm may promote prefrontal activation through multisensory integration mechanisms (Zatorre et al., 2007). The combination of auditory and motor processing increases the complexity of sensory input while optimizing the functionality of brain regions involved in cross-modal integration, such as the RFPA. Prior research has shown that music can enhance cognitive performance by stimulating the central nervous system and strengthening brain regions related to attention and memory (Särkämö et al., 2013; Thaut et al., 2015).

From the perspective of neural adaptation, long-term street dance training may enhance synaptic plasticity and improve the efficiency of neural networks, particularly in regions responsible for managing complex cognitive tasks. Evidence indicates that physical exercise stimulates the secretion of brain-derived neurotrophic factor (BDNF), which promotes neuroplasticity associated with learning and memory (Erickson et al., 2011; Korkusuz and Top, 2021). Through this mechanism, street dance may strengthen the functional connectivity between the RDLPFC and RFPA, thereby improving information processing capacity under high cognitive load.

### 4.2 Stroop task: improvement in inhibitory control

In the Stroop task, the street dance intervention significantly increased HbO₂ levels in the right dorsolateral prefrontal cortex (RDLPFC) and the right frontopolar area (RFPA), indicating enhanced inhibitory control. The dorsolateral prefrontal cortex plays a central role in conflict monitoring and cognitive inhibition, while the right frontopolar area is closely linked to higher-order control functions (Periáñez et al., 2021; Çemç and Ağduman, 2024). Furthermore, the significant activation of the left inferior frontal gyrus (LIFG) suggests improved allocation of cognitive resources during semantic conflict resolution.

The behavioral findings complement these neural results: participants in the street dance group demonstrated significant improvements in both accuracy and reaction time, particularly under incongruent conditions, whereas the control group showed no comparable changes. These improvements provide behavioral evidence that street dance training enhances inhibitory control efficiency, thereby supporting the fNIRS findings.

Several mechanisms may account for these effects. Street dance requires participants to cope with multitasking interferences in real time—coordinating rhythm, body movements, and spatial transitions simultaneously. This places high demands on selective attention and conflict resolution, processes largely supported by the prefrontal cortex. The rhythm synchronization and complex physical movements also train participants to ignore irrelevant distractions and maintain focus on task-relevant goals. Over time, such repeated engagement may promote functional plasticity in the RDLPFC and RFPA, strengthening their role in conflict monitoring and inhibitory control (Yang et al., 2022; Natale et al., 2017).

Additionally, the significant activation of the RDLPFC may be partly related to improved emotional regulation induced by the combination of music and movement. Previous studies have shown that rhythmic exercise accompanied by music can positively influence mood and emotional state, which in turn enhances executive function performance (Kawasaki and Hayashi, 2022). Similarly, the LIFG activation may reflect more efficient processing of semantic conflict, as both language-related processing and rhythm synchronization in dance rely on overlapping cognitive pathways. This suggests that the integration of music and coordinated movement may contribute to improvements in inhibitory control observed in the Stroop task.

### 4.3 More-odd shifting task: enhancement in cognitive flexibility

During the Shifting task, post-intervention HbO₂ levels in the right dorsolateral prefrontal cortex (RDLPFC) and the right frontopolar area (RFPA) significantly increased, indicating substantial improvements in participants’ task-switching ability and cognitive flexibility. The RDLPFC, as a core brain region for cognitive control, plays a critical role in rule switching, task transitions, and the execution of new task rules (Biro et al., 2021; Wang et al., 2024). The significant activation of the RFPA reflects participants’ enhanced ability for attentional resource reallocation and cognitive control during task transitions. Additionally, the increase in HbO₂ levels in the left inferior frontal gyrus (LIFG) further suggests that the street dance intervention improved participants’ ability to flexibly inhibit task rules and process semantic memory.

These neural findings were paralleled by behavioral improvements. The street dance group exhibited a marked increase in switching accuracy (+14.41%) and a significant reduction in reaction time (−109.88 ms), while also showing notable improvements in the non-switch condition (+5.06% accuracy, −77.06 ms reaction time). In contrast, the control group demonstrated smaller or non-significant improvements. This consistency between behavioral performance and prefrontal activation patterns underscores the ecological validity of the observed effects, confirming that street dance enhanced both neural efficiency and cognitive outcomes related to flexibility.

The enhancement of cognitive flexibility through the street dance intervention is likely linked to the multitask coordination demands inherent in dance training. Street dance requires participants to rapidly shift attention, dynamically adjusting movements, rhythm, and formations in response to complex and constantly changing environments (Kattenstroth et al., 2011). Such demands impose high cognitive loads on task-switching abilities, potentially promoting neurofunctional adaptability in the RDLPFC and RFPA over time. Participants’ need to flexibly switch between different task rules, similar to the demands of the Shifting task, provides a plausible explanation for the improvements observed (Rehfeld et al., 2017). Moreover, the frequent switching of rhythm and movement rules may effectively stimulate the functional development of the LIFG, strengthening its role in semantic processing during rule transitions.

The activation of the RFPA may also reflect the combined effects of attentional control and emotional regulation. Previous studies suggest that exercise interventions involving music can enhance executive function by improving mood and emotional states, thereby indirectly supporting performance in demanding task-switching contexts (Schibli et al., 2023).

Across all three tasks, it is also important to acknowledge that many prefrontal channels showed no significant changes, and in some cases only borderline effects (0.05 < *p* < 0.10) were observed. These findings should be interpreted with caution, as they may reflect potential but not robust intervention effects. Reporting both significant and non-significant outcomes helps delineate the regional specificity of the intervention and reduces the risk of selective reporting bias. Future studies with larger sample sizes are needed to clarify whether such borderline results represent meaningful trends or statistical variability.

### 4.4 Implications and future directions

This study demonstrates that street dance interventions can positively influence multiple core components of executive function, including working memory, inhibitory control, and cognitive flexibility. While aerobic exercise and other dance forms have also been shown to improve cognitive function, the present findings highlight the unique advantages of street dance. Characterized by strong rhythmicity, complex choreography, rapid spatial transitions, and improvisational demands, street dance imposes higher cognitive loads on working memory and attentional flexibility. Consequently, compared with less cognitively demanding forms of exercise, street dance may more effectively promote prefrontal activation and lead to significant improvements across multiple dimensions of executive function.

Future research could extend these findings in several ways. Direct comparisons across different dance styles would help clarify the unique role of street dance in enhancing cognitive function. Expanding the intervention to different genders and age groups could also provide insights into how sex and developmental stage moderate its effects. Additionally, longitudinal follow-up studies are needed to determine whether the benefits of street dance are sustained or even accumulate over time, thereby offering a deeper understanding of its contribution to cognitive reserve.

## 5 Conclusion

Studies have shown that an 18-week street dance intervention effectively improves working memory, inhibitory control, and cognitive flexibility, contributing to enhanced cognitive reserve. As a physical activity that integrates rhythm and movement coordination, street dance offers an early intervention strategy for delaying cognitive decline and reducing the risk of dementia.

## Data Availability

The raw data supporting the conclusions of this article will be made available by the authors, without undue reservation.
